# Supplementary material for: The impact of physical exercise on adolescents’ social-emotional competence: The chain mediating role of social support and peer relationships
Source: PLoS One. 2025 Nov 19;20(11):e0334587. doi: 10.1371/journal.pone.0334587 (PMC12629442; doi:10.1371/journal.pone.0334587)
Supplement: Attachment — Submitted filename: Response to Reviewers.docx [file pone.0334587.s003.docx]

Dear Prof. Elena del Pilar Jim&eacute;nez-P&eacute;rez; and all reviewers,

We sincerely thank you for your thoughtful and constructive feedback on our manuscript, "The Impact of Physical Exercise on Adolescents' Social-Emotional Competence: The Chain Mediating Role of Social Support and Peer Relationships" (Manuscript Number: PONE-D-25-20277). We greatly appreciate the time and attention you have dedicated to reviewing our work, and your insights have been instrumental in guiding our revisions. We have carefully considered each comment, implementing corresponding adjustments that we believe significantly enhance the rigor and clarity of our research. I will address the issues raised by the editor one by one in the cover letter. The following are detailed responses to the questions raised by all the reviewers:

**Reviewer 1**

**1.** This study aims to explore the relationship between physical exercise and adolescent socio-emotional competence and to analyze the mediating effects of social support and peer relationships in the chain. An interesting idea, but not a new one. This manuscript could bring an additional novelty, if it presented the details of the research application correctly and completely.

**Response:**

We sincerely appreciate your recognition of this research topic. Although it is not particularly novel, we consider it a vital direction for continued exploration in this field. Furthermore, regarding your suggestion that methodological details should be accurately and comprehensively presented, we fully endorse this perspective. In response to your feedback, we have thoroughly addressed this point in subsequent revisions, placing emphasis on refining and clearly presenting the applied research details with precision.

**2.** In the Introduction section, I recommend these studies, even though they are applied to athletes, most of whom are adolescents and provide support for the authors' argument:
<https://doi.org/10.1177/00315125211005235>, <https://doi.org/10.3390/children9050753>
<https://doi.org/10.7717/peerj.12803>, <https://doi.org/10.1177/00315125221135669>
<https://doi.org/10.3390/ijerph19031696>

**Response:**

①The article "Out-of-School Sports Participation Is Positively Associated with Physical Literacy, but What about Physical Education? A Cross-Sectional Gender-Stratified Analysis during the COVID-19 Pandemic among High-School Adolescents" proposes that "adolescents' engagement in physical exercise enhances their physical literacy, which encompasses physical, emotional, social, and cognitive attributes." This directly substantiates our assertion that "through physical exercise participation, adolescents improve physical literacy, thereby influencing self-awareness development" (Lines 50-51).

②The study "Measuring Perceived Social Support in Elite Athletes: Psychometric Properties of the Romanian Version of the Multidimensional Scale of Perceived Social Support" demonstrates that "social support promotes adolescents' sports participation." This empirically validates our proposed mechanism wherein "social support facilitates adolescents' engagement in physical exercise, ultimately forming a virtuous cycle" (Lines 278-279).

**3.** L151: "A total of 427 questionnaires were distributed ...." ??? Were 427 questionnaires or a single questionnaire applied to 427 subjects? I don't think it would be possible to apply 427 questionnaires!!! I think it's a mistake in the authors' expression. I recommend correct adaptation everywhere in this section and throughout the manuscript. Example here too &rdquo; 316 valid questionnaires&rdquo;???

**Response:**

We appreciate your identification of this ambiguity. Due to imprecise wording in our original description, we have revised the section as follows: (Lines 155-158):

A total of 427 integrated questionnaires containing four scales were distributed, yielding 427 responses. After excluding 111 invalid responses (e.g., incomplete or duplicate entries), 316 valid datasets were retained, representing a 74% validity rate.

**4.** L154 &rdquo;The sample included 164 males and 152 females...&rdquo; &ndash; I think this formulation should be adapted (example, maybe: teenagers and teenage girls), because at the age of 14 subjects cannot be classified as men or women

**Response:**

We sincerely appreciate your valuable feedback on the manuscript's phrasing. After consulting relevant literature, we fully concur with your perspective and have implemented the suggested revision:

Revised Statement (Line 158):"...including 164 boys (51.90%) and 152 girls (48.10%)."

**5.** L155: &rdquo; &plusmn; 1.405 years.&rdquo; ?? I think expressing withonly 2 decimal places would be ok! That is &rdquo; &plusmn; 1.40 years.&rdquo;

**Response:**

We gratefully acknowledge your insightful suggestion. The text has been revised as follows:

Revised Statement (Line 158): The participants' mean age was 14.73 ± 1.40 years.

**6.** I can't find the criteria for inclusion and exclusion of subjects in the study? I recommend highlighting them in the manuscript

**Response:**

We appreciate the reviewer's suggestions. In response to your recommendations, we have added detailed inclusion and exclusion criteria for the study participants in Section " 2.1 Participants". Revised Section " 2.1 Participants" (Lines 153-155):

The inclusion criteria for subjects were as follows: (1) Students aged 10–19; (2) Physically healthy with no motor impairments; (3) No cognitive impairments and able to accurately understand questionnaires and instructions. Individuals not meeting these criteria were excluded.

**7.** L163: &rdquo; Physical Activity Rating Scale developed by Hashimoto(1990)&rdquo; &ndash; why such an old instrument? 35 years old? The authors should argue, because today's adolescents have a completely different vision of social aspects, compared to respondents 35 years ago, when was the instrument validated? Was it validated on a specific population? (another question addressed to the authors)

**Response:**

We appreciate your question. Here is our detailed response to the issues raised:

①Regarding whether today's adolescents have a different perception of society compared to adolescents 35 years ago: We would like to clarify that the questions in the scale are relatively objective in nature, such as the intensity, duration, and frequency of physical activity. Therefore, differences in social perception would not affect the objectivity of the responses.

②Regarding whether the scale has been validated for specific populations: We confirm that the scale has been extensively validated for the adolescent population in numerous studies. We have also added the following content to the manuscript to provide further support: "Previous studies have demonstrated the applicability of this scale for adolescents [60, 61]."

The revised content is located in Lines 179-180 of the manuscript. Please review it.

**References：**

60.	Guo W, Shao W, Guo Y, Zheng L. Effect of HAES intervention on obese adolescents' eating disordertendency and physical exercise behavior: The mediating effect ofweight self-stigma. Journal of Physical Education. 2024;31(05):62-7. doi: 10.16237/j.cnki.cn44-1404/g8.2024.05.015.

61.	Guo Y, Chen Z, Wang H, Fan H. The Two-way Relationship Between Physical Activity and CognitiveReappraisal:A Study Based on Long-term Tracking and Diary Studies. Journal of Beijing Sport University. 2025;48(05):120-9. Doi : 10.19582/j.cnki.11-3785/g8.2025.05.010.

**8.** In the "2. Research Methods" section, data should be provided regarding the duration of completion, where were these assessment instruments applied, as a location? How long did it take to complete them? Were the subjects assisted by an adult? Did the subjects understand, accurately, at their age, all the items that the 4instruments assess? Did they have any questions and to whom did they address them? How were the 4 instruments applied: in a single form or in separate forms? Were the subjects aware that they were completing 4 assessments or did they know that they were completing a single instrument with 4 aspects? Is this section unclear and does not provide criteria for the reproducibility of the study?

**Response:**

We appreciate your identification of the issues. After carefully reviewing the "2. Research Methods" section, we have identified the deficiencies and have made the following point-by-point revisions:

①Regarding the data collection time: We have added the following information: Adolescents were surveyed between November 1 and November 30, 2024. (Lines 152-153)

②Regarding the location where the questionnaire was filled out: Paper questionnaires were administered by the research team during self-study sessions in classroom settings. (Lines 159-160)

③Regarding the estimated time to complete the questionnaire: an estimated completion time of 10 minutes. (Line 164)

④Regarding whether participants received assistance from adults and how their questions were addressed: We have added the following information: Before completing the questionnaires, researchers thoroughly explained the purpose, provided clear instructions, and addressed participants’ questions or concerns. Therefore, participants did receive adult assistance and had their questions addressed. (Lines 160-162)

⑤Regarding whether participants could accurately understand the questions posed by the four scales: We have added the following information:（1）The four questionnaires were selected from authoritative sources appropriate for the age group and have been validated for good reliability and validity among adolescents in this age range. We have also included references for each scale's application in adolescent populations in recent years in the"2.2 Variable Measurement" section. （2）The participants are normally educated adolescents aged 10-19 years, who possess the cognitive ability and logical thinking skills to accurately understand the questions posed in the questionnaire.(Lines 179, 185, 192, and 199)

⑥Regarding the format of the four scales: We have clarified the following information: Subjects were required to complete an integrated questionnaire comprising four scales: The Physical Activity Rating Scale, The Adolescent Social Support Scale, The Student Peer Relationships Scale, and The Social-Emotional Competence Questionnaire, totaling 66 items with an estimated completion time of 10 minutes. (Lines 162-164)

⑦ Regarding the provision of standards for research reproducibility: We have added the following information: This study has detailed the application of the research, including the inclusion and exclusion criteria for participants, detailed questionnaire information, experimental data, and the entire research process, which may provide a basis for verifying the experimental results.

We hope these revisions address your concerns.

**9.** The supplementary documents are not in English, which does not allow the reviewers to analyze these aspects (for example, I would like to find out details about Research protocols and Data.xlsx to verify the validity of the data, but I do not know the language used by the authors). I believe that translating the supplementary documents into English is essential, especially since some reviewers do not know the language used by the authors. I do not see the point of sending the main manuscript in English, the platform asks me if the English used by the authors is ok, but I, as areviewer, cannot analyze most of the documents!!!!!

**Response:**

We appreciate your feedback. We apologize for the oversight that led to some documents not being converted to English format, which may have caused inconvenience during the review process. We have now re-uploaded the research protocol and data in English on the system.

**10.** This study does not allow for data validation or reproducibility... due to the lack of certain details and supporting documents in a language that reviewers are not required to know in this journal.

**Response:**

We appreciate your feedback. We apologize for the oversight that led to some documents not being converted to English format, which may have caused inconvenience during the review process. We have now re-uploaded the data in English on the system.

**Reviewer 2**

**1.** The Introduction section should include more aspects of social support through sports activities.

**Response:**

We appreciate your question. In response, we have added relevant content regarding the acquisition of social support through physical exercise in the Introduction section. We have also included statements about the chain mediation model of physical exercise → social support → peer relationships → social and emotional competence. The revised content is as follows:

One study showed that physical exercise can help adolescents obtain social support from important figures such as friends and family, and promote the positive development of peer relationships [18]. Furthermore, the development of peer relationships was proven to be positively correlated with social-emotional competence, and this relationship is more pronounced among adolescent groups [19]. (Lines 54-57)

**References：**

19.	Weiss MR, Duncan SC. The relationship between physical competence and peer acceptance in tie context of children's sports participation. Journal of Sport and Exercise Psychology. 1992;14(2):177-91.

20.	Han X, Li H, Niu L. How does physical education influence university students’ psychological health? An analysis from the dual perspectives of social support and exercise behavior. Frontiers in Psychology. 2025;16:1457165

**2.**  I ask the authors to argue the relevance of the instruments used, compared to other instruments for evaluating the aspects targeted, which are newer, more recent (The first evaluation instrument is from 1990!!);

**Response:**

We appreciate your question. The scales we have selected have been widely cited in recent years and have undergone extensive validation among adolescent populations, demonstrating high reliability and validity. This indicates that the chosen scales possess a high level of authority. Additionally, in Section"2.2 Variable Measurement," we have included references for each scale cited within the past two years.

The supplementary content is located in Lines 179, 185, 192, and 199 of the manuscript. Please review it and let us know if you are satisfied.

**3.** I recommend that the authors add to the manuscript the criteria for including and excluding subjects in the research group

**Response:**

We appreciate the reviewer's suggestions. In response to your recommendations, we have added detailed inclusion and exclusion criteria for the study participants in Section " 2.1 Participants". Revised Section " 2.1 Participants" (Lines 153-155):

The inclusion criteria for subjects were as follows: (1) Students aged 10–19; (2) Physically healthy with no motor impairments; (3) No cognitive impairments and able to accurately understand questionnaires and instructions. Individuals not meeting these criteria were excluded.

**4.** I ask the authors to providemore details in the Research Methods section about how, when, where, how long the practical intervention lasted? (application of the questionnaire or the 4 evaluation instruments). To specify whether the adolescent subjects knew that they would be part of a research that targets several aspects!? The level of education of the adolescents is relevant and should be specified (were all respondents active in educational institutions?)

**Response:**

We appreciate your comments. In response to the issues you raised, we have carefully reviewed the “2. Research Methods” section and identified the deficiencies. We have made the following point-by-point revisions:

①Regarding the survey implementation method: We have added the following information: This study distributed one integrated questionnaire (containing four scales) in paper form to each of the 427 adolescent participants and excluded invalid questionnaires according to the screening criteria. (Lines 155-164)

②Regarding the survey time: We have added the following information: Adolescents were surveyed between November 1 and November 30, 2024. (Lines 152-153)

③Regarding the survey location: We have added the following information: The survey was conducted in three high schools in Guangdong Province, China, paper questionnaires were administered by the research team during self-study sessions in classroom settings. (Lines 159-160)

④Regarding whether adolescents were aware of the purpose of filling out the questionnaire:(1) We have added the following information: Written consent was obtained from the participants and their guardians before conducting the survey. (2) We have also added the following content to the manuscript: Before completing the questionnaires, researchers thoroughly explained the purpose, provided clear instructions, and addressed participants’ questions or concerns. (Lines 160-162)

⑤Regarding the educational level of the adolescents: We have added the following information: 108 senior high school students (34.18%) and 208 junior high school students (65.82%). (Lines 158-159)

We hope these revisions address your concerns.

**5.** How were the questionnaires administered to the over 300 subjects? Did the authors administer them and provide details, or were they assisted by other people? Were those people able to provide additional information, as clearly as the authors?! Why do I ask this? Because in the &ldquo;Acknowledgments&rdquo; one of the authors thanks colleagues and students who helped? Or are these &ldquo;students&rdquo; the subjects involved in the study?!! In this case, they should use the same term, &ldquo;adolescents&rdquo; everywhere! I ask the authorsto clarify!

**Response:**

We appreciate your questions. These issues also pertain to the "2. Research Methods " section. We will address them point by point as follows:

①Regarding how the questionnaires were distributed to the participants: We have added the following information: The questionnaires were not solely distributed by the authors themselves. Instead, the authors trained the research team to provide participants with accurate and consistent information. The added content is as follows: Before conducting the questionnaire survey, to standardize data collection, all researchers received centralized training in advance. During fieldwork, they monitored participants’ completion process. (Lines 165-166)

②Regarding the content of the "Acknowledgements" section: The confusion arose due to imprecise wording. The students thanked in the "Acknowledgements" section are the adolescents who participated in the survey. We have revised the wording as suggested, changing the term to "adolescents". We hope these revisions address your concerns.

**6.** Were the adolescents involved in the research provided with information regarding their role in what they have to complete or answer?

**Response:**

We appreciate your question. In response to this issue, we have provided the following reply:

(1) Written consent was obtained from the participants and their guardians prior to conducting the survey. (2) We have also added the following content to the manuscript: Before completing the questionnaires, researchers thoroughly explained the purpose, provided clear instructions, and addressed participants’ questions or concerns. (Lines 160-162)

Therefore, the adolescents who participated in the study had a full understanding of their role in the research process and the specific tasks they were required to complete before filling out the questionnaire.

**7.** Were the assessment tools validated on the population, of which the subjects of this study are part?

**Response:**

We appreciate your question. The scales we have selected have been widely cited in recent years and have undergone extensive validation among adolescent populations, demonstrating high reliability and validity. Additionally, in Section"2.2 Variable Measurement", we have included references for each scale cited within the past two years at the end of the description for each scale.

The supplementary content is located in Lines 179, 185, 192, and 199 of the manuscript.

**8.** I think that the numerical data in the manuscript should all be in the same format (with two decimal places). Please check everywhere in the manuscript

**Response:**

We sincerely appreciate your valuable suggestions. Based on your recommendations, we have carefully reviewed the content of the manuscript and revised the data format to retain two decimal places.

**9.** If the study is about children / adolescents, I recommend eliminating the words "male", "female"

**Response:**

We sincerely appreciate your valuable comments on the rigor of the terminology used in this manuscript. We fully agree with your perspective that the expressions you pointed out are indeed significant oversights. We have revised the terminology regarding males and females to boys and girls, respectively. The specific revision is as follows: including 164 boys (51.90%) and 152 girls (48.10%). （Line 158）

**10.** In table 2, p=0.000.??? cannot be checked because the additional documents are not in English

**Response:**

We appreciate your feedback. We apologize for the oversight that led to some documents not being converted to English format, which may have caused inconvenience during the review process. We will now re-upload the data in English on the system.

**11.** I did not clearly understand what the purpose of the research was (the authors should clarify): to highlight certain relationships between the components of social support or to build a model and propose this model? I ask the authors to clarify in the conclusions and at the end of the discussions

**Response:**

We appreciate the reviewer's suggestions. In response to the issues you raised, we have added the following content to the "Conclusion" section: This study constructed a chain mediation model through a cross-sectional survey of adolescents in Guangdong Province, China, to investigate the relationship between physical exercise and adolescents' social-emotional competence. It provides theoretical support and practical evidence for cultivating adolescents' social-emotional competence.

The supplementary content is located in Lines 340-342 of the manuscript.

**12.**  I believe that the limitations of the study are few and I recommend the authors to analyze all aspects and specify them clearly (the study, as it is proposed, does not provide enough details to be replicated, due to limitations of context, age, level of education of the respondents, etc. etc.)

**Response:**

We appreciate your question. In response to the issues you raised, we have added the following content to the "Limitations" section: this study sampled adolescents from Guangdong Province, China. Due to influences from their educational and social contexts, the findings’ applicability across diverse backgrounds may be limited. Subsequent research should be conducted in multiple countries and varied settings.

The supplementary content is located in Lines 336-338 of the manuscript.

**13.** The conclusions are presented schematically!! I think it is more academic for authors to formulate clearly, concisely and scriptively what the ideas that resulted are. If readers want to read the title, abstract and conclusions, to see the basic idea, they have nothing to understand in the conclusions, in the form in which they are presented. The scheme with arrows can be included in the discussions or in the form of diagrams or figures, but the conclusions must be written

**Response:**

We appreciate your question. In response, we have revised the "Conclusion" section to use more concise wording and avoid the use of bullet points or list format. The revised content is as follows: This study constructed a chain mediation model through a cross-sectional survey of adolescents in Guangdong Province, China, to investigate the relationship between physical exercise and adolescents' social-emotional competence. It provides theoretical support and practical evidence for cultivating adolescents' social-emotional competence, yielding the following conclusions:(1) Physical exercise can significantly and positively predict adolescents' social-emotional competence;(2) Social support and peer relationships play a chain mediating role between physical exercise and adolescents' social-emotional competence.

The revised content is located in Lines 343-345 of the manuscript.

**14.** In References, there are extremely many references from the Asian area, which could provide subjectivity to the argued aspects!! It is not a bad thing, but it orients the ideas towards a certain area of the world, with certain influences of education or thinking, which does not offer the possibility of generalizing the conclusions. If they still maintain it, then the authors should conclude with reference to a certain typology of population. Maybe even in the title the reference to teenagers from China appears, and then the purpose and discussions that orient the idea are much clearer.

**Response:**

We sincerely appreciate your valuable comments. We fully agree with your perspective. Since the study population of this research was selected from adolescents in Guangdong Province, China, we might have tended to choose literature with similarly backgrounded subjects during the literature review, which led to an overrepresentation of references from the Asian region. To address this, we have added supplementary information in both the "Limitations" and "Conclusion" sections of the paper.

① Supplementary content in the "Limitations" section:

this study sampled adolescents from Guangdong Province, China. Due to influences from their educational and social contexts, the findings’ applicability across diverse backgrounds may be limited. Subsequent research should be conducted in multiple countries and varied settings. (Supplementary content located in Lines 336-338)

② Supplementary content in the "Conclusion" section:

This study constructed a chain mediation model through a cross-sectional survey of adolescents in Guangdong Province, China, to investigate the relationship between physical exercise and adolescents' social-emotional competence. It provides theoretical support and practical evidence for cultivating adolescents' social-emotional competence. (Supplementary content located in Lines 340-342)

We will also avoid making overly broad generalizations that exceed the scope supported by the data.

**15.** How did the authors evaluate whether each question is relevant, clear and sufficient?

**Response:**

We sincerely appreciate your question. The scales we have selected have been widely cited in recent years and have undergone extensive validation among adolescent populations, demonstrating high reliability and validity. This indicates that the items in each scale are correlated and that the wording of the questions is clear and comprehensive. Additionally, in Section"2.2 Variable Measurement", we have included references for each scale cited within the past two years at the end of the description for each scale.

The supplementary content is located in Lines 179, 185, 192 and 199 of the manuscript. Please review it and let us know if you are satisfied.

**16.** How were the items translated or adapted for the subjects? Who checked the validity of the translation?

**Response:**

We appreciate your question. In response to the issues regarding the applicability and translation validity of the scales, we provide the following reply: The scales used in this study were either developed or revised by previous researchers in Chinese. These scales have been proven to have good reliability and validity during their development or revision processes and have been demonstrated to be applicable to adolescents in previous studies. Therefore, in this study, there is no involvement of translation or adaptation. Please review this response.

**17.** I recommend carefully checking the references, some are not relevant to this article, because they do not target the same age or context, and others are on subjects with conditions, and the authors did not specify anything about this aspect regarding the subjects in this study!!!

**Response:**

We appreciate your question. In response, we have carefully reviewed the "References" section and identified that some of the cited studies had specific background limitations that are not relevant to the context of our study. To address this, we have replaced eight references that were not consistent with the background of our study subjects. These changes have been highlighted in the manuscript.

The revised references are located in the "References" section at items 15, 27, 39, 41, 48, 54, 90 and 101. Please review these changes

**Reviewer 2**

**1.** The article begins by mentioning the importance of "non-cognitive skills", but its relationship to "social-emotional skills" is unclear. It is recommended to add a transition sentence.

**Response:**

We appreciate your question. Due to an error in our wording, the meaning was misinterpreted. We have revised the statement in the main text as follows: Social-emotional competence serving as a core assessment indicator of non-cognitive skills, attracts continuing research attention from the international academic community.

The revised content is located in Lines 26-27 of the manuscript. Please review it.

**2.** The third paragraph mentions the definition and impact of physical exercise, but suddenly inserts "Scientific participation in physical exercise..." This seems abrupt. It is recommended to make a clear transition in the previous sentence.

**Response:**

We appreciate your feedback. Based on your suggestions, we have revised the content by removing the description regarding "scientific participation in physical exercise..." to make the expression more reasonable.

The revised content is located in Lines 48-51 of the manuscript. Please review it.

**3.** The citation format is inconsistent, some have spaces before the brackets, and some do not.

**Response:**

We sincerely appreciate your valuable comments on the formatting rigor of this manuscript. We have carefully reviewed the format and confirmed that, according to the English manuscript layout standards, there should be a space before the parentheses in citations. We have proofread the entire paper and revised it to a uniform format to ensure that such issues will not occur again.

**4.** There are issues with the standardization of English punctuation marks. Some punctuation marks have no space after them and need to be fully checked.

**Response:**

We sincerely appreciate your valuable comments on the formatting rigor of this manuscript .We have carefully reviewed the format and confirmed that, according to the English manuscript layout standards, a space should be added after punctuation marks. We have proofread the entire paper and revised it to a uniform format to ensure that such issues will not occur again.

**5.** The overall research design is too simple, the method is outdated, and lacks innovation.

We appreciate your comments.In response,we provide the following reply:

**Response:**

Although cross-sectional studies cannot determine causality, their efficiency allows us to validate the associations between key variables within limited resources, providing a basis for subsequent longitudinal studies. Moreover, this study employs the classic framework of chain mediation, for the first time revealing the chain mediation effect of social support and peer relationships between physical exercise and adolescents' social and emotional competence. This offers a new perspective for cultivating adolescents' social and emotional competence. Finally, we have emphasized the supplementation and improvement of the application details of this study to more accurately present its content.

Therefore, this study is not entirely simple, outdated, or lacking in innovation. Additionally, we will refer to cutting-edge international research in future studies to further improve this research. We hope this response is satisfactory.

**6.** The article mentioned the distribution and return of questionnaires, but did not describe the sampling method (e.g., random sampling, convenience sampling), which affects thereproducibility of the study.

**Response:**

We appreciate your question. In response to the lack of description regarding the sampling method, we have added the following content: This study employed a convenience sampling method. The revised content is located in Line 151 of the manuscript.

**7.** The calculation (Duration &times; Intensity &times; Frequency) does not indicate whether it has been validated or whether other studies support the rationality of the formula.

**Response:**

We appreciate your question. In response, we provide the following reply: Since its development, the scale has been widely cited and has become an authoritative questionnaire for measuring physical activity levels. It has also undergone extensive validation among adolescent populations, demonstrating high reliability and validity. This indicates that the rationality of the formula has been repeatedly verified. Please review this response and let us know if you are satisfied.

**8.** The number of bootstrap samples (usually 5000) and the type of confidence interval (e.g., bias-corrected 95% CI) were not specified.

**Response:**

We appreciate your feedback. In response to the lack of description regarding this part of the content, we have added the following information in the"3.3 Mediating Effects of Social Support and Peer Relationships" section: a total of 5,000 Bootstrap samples were used to test the mediating effects, generating bias-corrected (BC) 95% confidence intervals.

The supplementary content is located in Lines 229-231 of the manuscript.

**9.** "Unrotated exploratory factor analysis extracted 11 factors with eigenvalues >1, the largest of which explained 34.719% of the variance (<40%)." It was not stated whether principal component analysis (PCA) or exploratory factor analysis (EFA) was used, and the method was vague.

**Response:**

We appreciate your feedback. In response to the lack of description regarding this part of the content, we have added a description of EFA in the"3.1 Common Method Bias Test" section.

The supplementary content is located in Lines 212-213 of the manuscript.

**10.** 3.2 Descriptive statistics and correlation analysis Only significance (p < 0.01) was reported, and the strength of the correlation coefficient (r) was not stated, which suggests that itis additional.

**Response:**

We appreciate your feedback. In response to the lack of description regarding this part of the content, we have added the following information: Among these, social support shows the strongest correlation with social-emotional competence (r = 0.72). Peer relationships exhibit the second-highest correlation with social-emotional competence (r = 0.51), while their correlation with social support is relatively weaker (r = 0.42). Physical exercise displays moderately positive correlations with social-emotional competence (r = 0.43), peer relationships (r = 0.38), and social support (r = 0.30). These data provide preliminary evidence suggesting that social support and peer relationships may play mediating roles in the relationship between physical exercise and adolescents’ social-emotional competence.

The supplementary content is located in Lines 219-225 of the manuscript.

11. The font size of the table is not uniform, and it is recommended to coordinate the reference format.

**Response:**

We appreciate your feedback. Initially, we chose different font sizes for the tables to enhance their visual appeal. However, we will follow your suggestion and adjust the table fonts to a uniform size.

**12.** 4.1 Physical Exercise and Social-Emotional Competencies

&ldquo;Physical exercise plays a key role in improving adolescents&rsquo; social-emotional competences. Cross-sectional studies cannot prove causality and cautionary statements should be used.

**Response:**

We sincerely appreciate your valuable comments on the rigor of the terminology used in this manuscript. We fully agree with your perspective that the imprecise wording led to the error. In response, we have removed the result-oriented description and revised the content to: Therefore, physical exercise can significantly and positively predict adolescents' social-emotional competence. The revised content is located in Line 266 of the manuscript.

**13.** 4.2 Independent mediating role of social support

Literature comparison is weak and only cites &ldquo;previous findings&rdquo; without specifying which studies support them.

**Response:**

We sincerely appreciate your valuable feedback. After carefully reviewing the content in question, we identified that the section indeed lacked a basis for the internal mechanisms through which physical exercise provides social support. To address this, we have added supporting evidence for these research findings and revised the content as follows:

Adolescents from families that maintain long-term physical exercise habits typically have closer relationships with their parents and are more likely to receive greater familial support [80]. This occurs because adolescents' cognitive abilities are not yet fully developed, and their lower economic status within families makes parental involvement in family physical exercise more focused on conscious and deliberate assistance. Consequently, adolescents receive multidimensional family support, including instrumental support (e.g., sports equipment purchases, fitness investments), informational support (e.g., verbal instruction and behavioral modeling of physical activities), and appraisal support (e.g., encouragement and praise) [81, 82]. Furthermore, during physical exercise participation—particularly in team sports or competitive settings—adolescents strengthen the frequency and depth of peer interactions, building trust and belongingness with others [83]. This establishes a foundation for forming long-term interpersonal networks and provides sustainable social support [84], which in turn promotes adolescents' engagement in physical exercise, creating a virtuous cycle [85].

The revised content is located in Lines 269-279 of the manuscript. Please review it and let us know if you are satisfied.

**References：**

80.	Hu Y, Tang Y, Zhang J, Liu Y. Research of Parent Factors Associated with Adolescents’

Moderate-to-Vigorous Physical Activity. China Sport Science and Technology. 2017;53(03):14-21. Doi : 10.16470/j.csst.201703003.

81.	Yang A, He C. Relationship Between Family Physical Education and Students’ Mental Health in Compulsory Education Stage: Chain Mediating Effects of Parent-Child Communication and Exercise Behavior. Journal of Shenyang Sport University. 2024;43(03):15-22.

82.	Huang L. Preliminary Exploration of Social Support Theory. Psychological Monthly. 2020;15(16):238-9. Doi : 10.19738/j.cnki.psy.2020.16.221.

83.	Yu S, Liu B. The Origin of Sports Diplomacy: A Study Based on the Investigation of Political Modernity. Journal of Beijing Sport University. 2024;47(05):76-85. doi: 10.19582/j.cnki.11-3785/g8.2024.05.006.

84.	Lei H, Peng B, Tang M, Chen W, Wang H, Yu T. A study on the relationship between college students’ physical exercise and feelings of inferiority: The mediating effect of social support. PLoS One. 2025;20(5): e0321685.

85.	Alexe DI, Sandovici A, Robu V, Burgueño R, Tohănean DI, Larion AC, et al. Measuring Perceived Social Support in Elite Athletes: Psychometric Properties of the Romanian Version of the Multidimensional Scale of Perceived Social Support. Perceptual and Motor Skills. 2021;128(3):1197-214. doi: 10.1177/00315125211005235.

**14.** &ldquo;Social support equips adolescents with basic social skills&hellip;&rdquo; Specific skills (e.g., conflict resolution, empathy) are not specified.

**Response:**

We appreciate your question. In response, we have added information regarding the specific social skills that social support can provide to adolescents(as follows): By earning recognition and support from family, school, and society through physical exercise, adolescents strengthen self-identity, refine social skills, this fosters prosocial behavior in adolescents [87] and enhances their empathic ability [88]

The revised content is located in Lines 282-283 of the manuscript. Please review it.

**References：**

87.	Chang J, Zhu H, Zhang X, Li C. Effect of social support on adolescents' prosocial behavior: mediating effect of social anxiety and depression. Modern Preventive Medicine. 2021;48(12):2210-3+67. doi: 10.20043/j.cnki.mpm.2021.12.019.

88.	Qi H, Yang H. The relationship between nursing iterns′empathy and their perceived social support. Chinese Clinical Nursing. 2020;12(04):377-9.

**15.** 4.3 Independent mediating role of peer relationships
The mixed use of "peer acceptance" and "peer relationships"; coordination is suggested. Classical theories (e.g., Sullivan's interpersonal relationship theory) can be cited to explain the role of peer relationships.

**Response:**

We sincerely appreciate your valuable comments on the rigor of the terminology used in this manuscript. We acknowledge the confusion between "peer acceptance" and" peer relationships" and have made the necessary revisions. We have replaced the two instances unrelated to "peer acceptance" with "peer relationships" to clarify the expression. Additionally, we have carefully reviewed Sullivan's interpersonal theory and cited it to explain the mechanism through which peer relationships influence social and emotional competence. The specific supplementary content is as follows: Sullivan's interpersonal relations theory regards peer relationships as a key factor in individuals' socialization development and healthy personality formation, playing a crucial role in the development of adolescents' self-awareness and interpersonal skills [92].

The revised content is located in Lines 300-302 of the manuscript. Please review it.

**References：**

92.	Sullivan HS. The interpersonal theory of psychiatry: Routledge; 2013.

**16.** 4.4 Chain mediation model analysis
"Social support consistently enhances peer relationships" Unexplained mechanism, how social support reduces peer conflict.

**Response:**

We appreciate your question. In response, we have added the mechanism through which social support can reduce peer conflict. The specific content is as follows: Higher levels of social support can further promote the development of peer relationships. For instance, familial support enhances functional integration of the prefrontal-limbic system, reducing sensitivity to peer rejection [56]. Teacher support reduces peer conflicts, as teachers provide timely and consistent responses to conflicts, spend time listening to both parties, and help adolescents resolve interpersonal conflicts [96].

The revised content is located in Lines 319-323 of the manuscript. Please review it and let us know if you are satisfied.

**References：**

63.	Guyer AE, Jarcho JM, Pérez-Edgar K, Degnan KA, Pine DS, Fox NA, et al. Temperament and parenting styles in early childhood differentially influence neural response to peer evaluation in adolescence. Journal of abnormal child psychology. 2015;43:863-74.

96.	Wang L-J, Wang W-C, Gu H-G, Zhan P-D, Yang X-X, Barnard J. Relationships among teacher support, peer conflict resolution, and school emotional experiences in adolescents from Shanghai. Social Behavior and Personality: an international journal. 2014;42(1):99-113.

**17.** 4.5 Limitations
No mention of sample representativeness (only three schools in Guangdong) and homology bias. "Considering other potential variables, such as cognitive reappraisal..." No explanation of the relationship of these variables to the existing model.

**Response:**

We sincerely appreciate your detailed and constructive review comments, which are crucial for us to further improve the manuscript. In response to your valuable suggestions, we have carefully reflected and plan to make the following revisions:

①Regarding the representativeness of the sample: Given the strict limitations of time, budget, and human resources, this study employed a convenience sampling method, which indeed limits the representativeness of the sample. However, we have detailed the demographic characteristics and data collection methods in Section" 2.1 Participants "which can help clarify the scope of application for the study conclusions. We have also addressed this issue in Section"4.5 Limitations."

②Regarding common method bias: At the outset of sample selection, we endeavored to consider the diversity of the sample. We have supplemented in Section" 2.1 Participants "that the sample was drawn from schools in different cities. During the data collection phase, we implemented procedural controls (such as anonymous completion and reverse scoring) and reported the results of the Harman single-factor test in Section"3.1 Common Method Bias Test" which indicated no significant common method bias.

③Regarding the connection between these variables and existing models: The misunderstanding arose due to imprecise wording. What we intended to convey is that whether there are other potential variables remains to be verified through subsequent research. The revised content is as follows: this study only examined the mediating effects of social support and peer relationships in the relationship between physical exercise and adolescents' social-emotional competence. However, whether other potential variables exist requires verification through subsequent research. The revised content is located in Lines 332-334 of the manuscript.

We hope these revisions address your concerns.
